# Performance of the Alere i RSV assay for point-of-care detection of respiratory syncytial virus in children

**DOI:** 10.1186/s12879-017-2855-1

**Published:** 2017-12-13

**Authors:** Sarah Valerie Schnee, Johannes Pfeil, Clara Marlene Ihling, Julia Tabatabai, Paul Schnitzler

**Affiliations:** 1 Center for Childhood and Adolescent Medicine (General Pediatrics), University Hospital Heidelberg, Im Neuenheimer Feld 420, 69120 Heidelberg, Germany; 2 German Center for Infection Research (DZIF), Heidelberg partner site, Heidelberg, Germany; 3 Center for Infectious Diseases, Virology, University Hospital Heidelberg, Heidelberg, Germany

## Abstract

**Background:**

Respiratory syncytial virus (RSV) is the most important cause of severe acute respiratory tract infection in young children. Alere i RSV is a novel molecular rapid test which identifies respiratory syncytial virus in less than 13 min.

**Methods:**

We evaluated the clinical performance of the Alere i RSV assay in a pediatric point-of-care setting during winter season 2016 / 2017. Test results from 518 nasopharyngeal swab samples were compared to a real-time reverse transcription PCR reference standard.

**Results:**

The overall sensitivity and specificity of the Alere i RSV test assay was 93% (CI^95^ 89% – 96%) and 96% (CI^95^ 93% – 98%), respectively. Alere i RSV performed well in children of all age groups. An optimal sensitivity of 98% (CI^95^ 94% - 100%) and specificity of 96% (CI^95^ 90% - 99%) was obtained in children < 6 months. In children ≥ 2 years, sensitivity and specificity remained at 87% (CI^95^ 73% – 96%) and 98% (CI^95^ 92% – 100%), respectively. False negative Alere i RSV test results mostly occurred in samples with low viral load (mean CT value 31.1; CI^95^ 29.6 – 32.6). The Alere i RSV assay is easy to use and can be operated after minimal initial training. Test results are available within 13 min, with most RSV positive samples being identified after approximately 5 min.

**Conclusion:**

The Alere i RSV assay has the potential to facilitate the detection of RSV in pediatric point-of-care settings.

**Electronic supplementary material:**

The online version of this article (10.1186/s12879-017-2855-1) contains supplementary material, which is available to authorized users.

## Background

Respiratory Syncytial Virus (RSV) is the most important cause of acute respiratory tract infection (aRTI) in neonates and young children worldwide [1]. RSV is a frequent cause of hospitalization in young children and leads to significant morbidity in premature neonates and children with chronic lung or congenital heart disease [2–4].

The clinical diagnosis of RSV is hampered by the mostly unspecific symptoms of RSV infection. Early recognition of RSV infection is useful to optimize care management, minimize unnecessary antibiotic use [5] and provide targeted infection control for children hospitalized with RSV infection [6]. In addition, specific antiviral therapy for RSV infection is currently under early clinical evaluation [7], and early detection of RSV infection may become important for timely antiviral treatment in severely sick children.

Pediatricians therefore often apply rapid point-of-care RSV test assays. The major limitation of point-of-care RSV testing is the low sensitivity of commercially available rapid antigen detection tests (RADT). RADT sensitivity is strongly dependent on viral load, and therefore performs best in young infants with classical symptoms of RSV bronchiolitis. In older children and adults with low viral load, the sensitivity is poor and RADT are not recommended in these age groups [8, 9].

Alere i RSV is a novel rapid molecular test assay which can identify RSV in less than 13 min. In a previous analysis, we reported an Alere i RSV sensitivity and specificity of 100% (CI_95_ 89–100%) and 97% (CI_95_ 89% – 100%), respectively [10]. This first analysis focused on young infants, and testing was done under laboratory conditions which may not reflect the performance in point-of-care settings.

In the current study, we addressed these limitations and applied the Alere i RSV test assay in a pediatric point-of-care setting on a larger study population across different pediatric age groups. The objective of this analysis was to report an estimate of the Alere i RSV test performance in a pediatric point-of-care setting.

## Methods

### Study cohort and sampling procedure

Between November 2016 and March 2017, we prospectively collected 533 nasopharyngeal swabs (NPS) in the outpatient department of the Center of Childhood and Adolescent Medicine Heidelberg, Germany. Study inclusion criteria were I) age < 18 years, II) clinical symptoms of an acute respiratory infection, and III) indication for hospitalization according to the clinical judgment of the attending physician. Patients with clinical symptoms of an acute respiratory infection included cases of upper respiratory tract infection (URTI), otitis media, croup, bronchiolitis, bronchitis and pneumonia.

Nasopharyngeal swabs were collected by local staff in 1 ml viral transport media (VTM; MSwab; Copan, Brescia, Italy). 200 μl of the VTM were directly used for point-of-care testing with the Alere i RSV assay. The remaining sample was transferred to the virology diagnostic laboratory, and stored in 200 μl aliquots at −80 °C until further analysis.

Attending pediatricians prospectively reported medical information on a standardized data sheet, including the duration of clinical symptoms, demographic and clinical data.

### Alere i RSV assay

Alere i RSV test assays were applied in the pediatric outpatient department. The test procedure followed the Alere i RSV package insert [11]. In brief, 200 μl of the respiratory sample was added to the sample receiver containing 2.5 ml elution buffer. Two 100 μl volumes were added to the test base with the provided transfer cartridge for isothermal amplification.

Alere i RSV assays were conducted by attending physicians or nurses working in the pediatric outpatient department. All operators were allowed to carry out the test assay only after an initial hands-on-training. Test results were printed and reported on the medical datasheet. Invalid test results were re-tested immediately.

### Reference standard (RT-PCR test procedures)

The Alere i RSV assay was evaluated against a CE-marked real-time reverse transcriptase polymerase chain reaction assay (RT-PCR).

For RT-PCR analysis, RNA was extracted from 140 μl respiratory specimen using the QIAamp® viral RNA mini kit (Qiagen, Hilden, Germany) according to the manufacturer’s protocol. Amplification and detection of viral RNA was performed by FTD respiratory pathogens 21 multiplex PCR (FTD 21, Fast-track diagnostics Ltd., Sliema, Malta) on a LightCycler® 480 instrument II (Roche, Mannheim, Germany). The FTD 21 assay can detect the following pathogens: influenza A, H1N1 or B, rhinovirus, respiratory syncytial virus, bocavirus, adenovirus, parainfluenza 1, 2, 3 or 4, coronavirus NL63, 229E, OC43 or HKU1, parechovirus, enterovirus, human metapneumovirus A/B and Mycoplasma pneumoniae.

FTD 21 results that did not correspond to the Alere i test assay were verified by a second RT-PCR assay. For this purpose, RNA was extracted from an independent sample aliquot and analyzed by altona RealStar PCR (altona RealStar RSV RT-PCR, altona Diagnostics, Hamburg, Germany). To preclude possible discrepancies between the two RT-PCR methods, samples with different results in monoplex and multiplex PCR were again tested by the FTD 21 assay (Additional file 1: Figure S3).

Both the FTD 21 and the altona RealStar assay were initially evaluated using defined RSV A and RSV B positive and negative samples from patients, from the German RSV reference laboratory and from the official German proficiency testing panel. Results with a cycle threshold (C_T_) value of <35 were considered positive.

For sub-typing of RSV positive samples, Sanger sequencing targeting the second hyper-variable region of the G gene was performed using primer pairs as previously described [12]. Resulting sequences were assembled and edited using the SEQMAN II software of the Lasergene package (DNAstar, Madison, WI) and allocated to sub-type RSV A or RSV B using the Basic Local Alignment Search Tool (BLAST; *http://blast.ncbi.nlm.nih.gov*).

### Statistical analysis and data reporting

Statistical analyses were conducted using Stata/IC13.0 (StataCorp. LP, College Station, TX, USA). Mann-Whitney-U-test was applied to compare C_*T*_ values in samples with true positive versus false negative Alere i RSV result. The Alere i RSV sensitivity in RSV A versus RSV B positive samples was compared using the χ^**2**^-Test. *P* values <0.05 were considered statistically significant. Data reporting was done according to STARD 2015 recommendations [13].

## Results

From November 2016 to March 2017, 533 NPS were collected from 499 children presenting with symptoms of acute respiratory tract infection. Fifteen samples were excluded from the final analysis due to unavailability of sample aliquots for RT-PCR testing (*n* = 7), duplicate sampling of patients during one hospital stay (n = 7), or missed re-testing of an initially invalid Alere i RSV test result (*n* = 1).

**Fig. 1:**
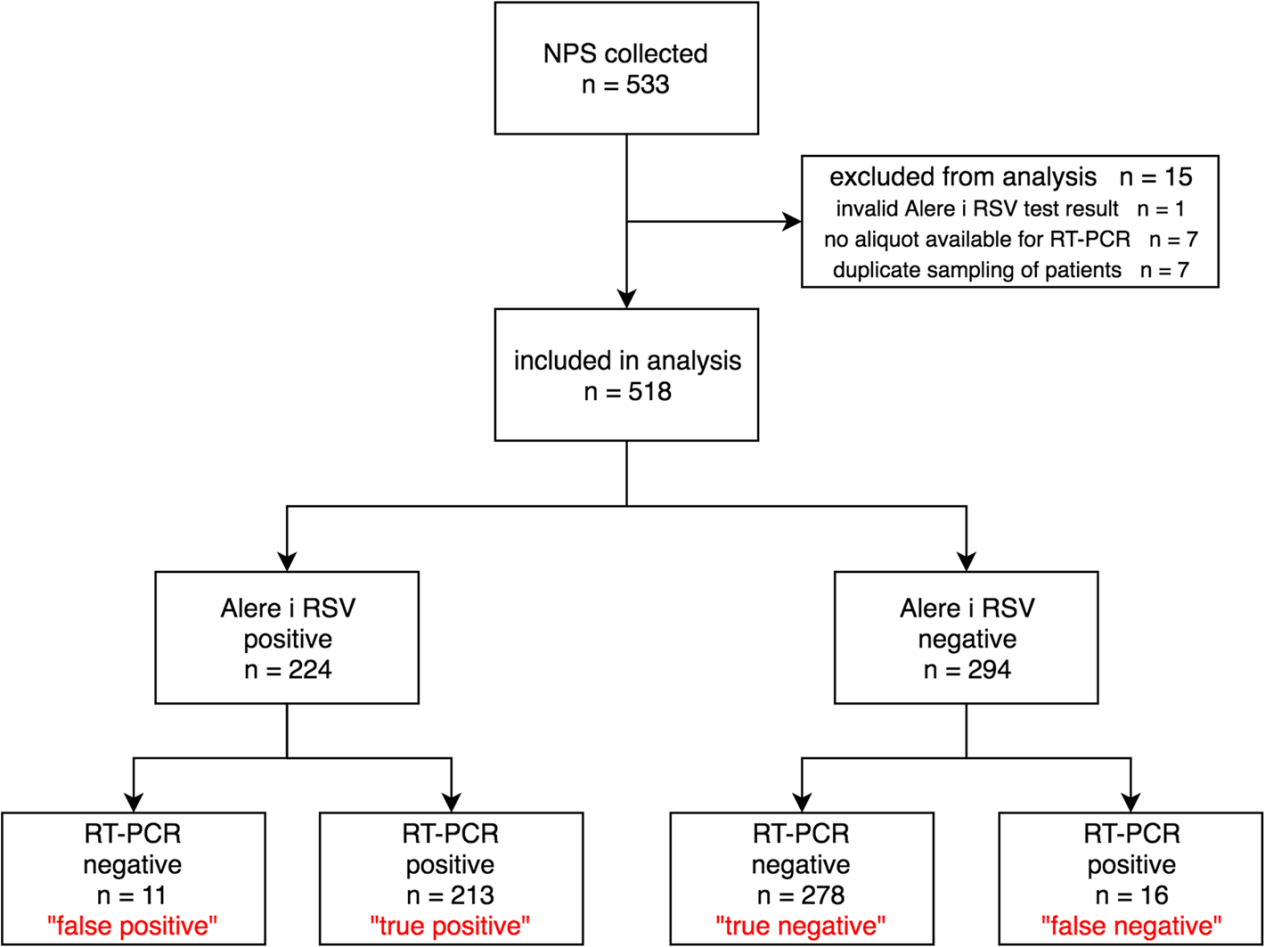
Study Flow Chart

Five hundred eighteen samples (97% of 533 collected NPS) from 492 children were included in the final analysis (Fig. 1). Demographic and clinical characteristics of the study participants are summarized in Table 1.

**Table 1:** Baseline demographic and clinical characteristics of 518 study participants

N = 518	n	% of total
gender		
male	292	57%
female	226	43%
age		
0–5 months	212	41%
6–11 months	76	15%
12–23 months	107	21%
≥ 2 years	123	24%
duration of clinical symptoms on admission		
≤ 3 days	276	53%
> 3 days	218	42%
unknown	24	5%
admission diagnosis		
URTI	81	16%
LRTI	257	49%
non-respiratory	180	35%

We grouped the admission diagnoses in cases of URTI (*n* = 81, including URTI, otitis media and croup), LRTI (*n* = 257, including bronchiolitis, bronchitis and pneumonia) and non-respiratory admission diagnoses (n  =  180). The latter includes children admitted for non-respiratory reasons (e.g. febrile convulsion, diarrhea) with concomitant acute RTI

Alere i RSV was positive in 43% (224/518) and negative in 57% (294/518). In comparison to the RT-PCR reference standard, the Alere i RSV test result was true positive in 213 and true negative in 278 samples, respectively. False positive test results were reported in 11 patients, and 16 patients were identified with false negative Alere i RSV test outcome (Table 2). The overall Alere i RSV test sensitivity and specificity was 93% (CI_95_ 89% – 96%) and 96% (CI_95_ 93% – 98%), respectively.

**Table 2:** Cross tabulation of Alere i RSV test results by the RT-PCR result

	Alere i RSV positive	Alere i RSV negative	total
RT-PCR positive	213	16	229
RT-PCR negative	11	278	289
total	224	294	518

**Table 3:** Alere i RSV test sensitivity and specificity by admission diagnosis and age group

	Alere i RSV test result		total	sensitivity in % (CI_95_)	Alere i RSV test result		total	specificity in % (CI_95_)
	true positive	false negative			true negative	false positive		
	213	16	229	93 (89–96)	278	11	289	96 (93–98)
admission diagnosis								
URTI	21	0	21	100 (84–100)	57	3	60	95 (86–99)
LRTI	159	6	165	96 (92–99)	89	3	92	97 (91–99)
non-respiratory	33	10	43	77 (61–88)	133	5	138	96 (92–99)
age group								
0–5 months	112	2	114	98 (94–100)	94	4	98	96 (90–99)
6–11 months	33	2	35	94 (81–99)	40	1	41	98 (87–100)
12–23 months	34	7	41	83 (68–93)	62	4	66	94 (85–98)
≥ 2 years	34	5	39	87 (73–96)	83	2	85	98 (92–100)

The mean FTD 21 *C*_*T*_ value of true positive samples was 17.7 (CI_95_ 17.1–18.3; range 11.7–34.6). NPS with false negative Alere i RSV result had a significantly higher mean *C*_*T*_ value of 31.1 (CI_95_ 29.6–32.6; range 25.4–34.5, *P* < 0.001; Mann-Whitney-U-test).

RSV A infection was detected in 65% (149 / 229) and RSV B in 32% (74 / 229) of RSV positive NPS. In two samples, both RSV A and B were identified. In 4 cases, no subtype could be determined. The sensitivity of the Alere i RSV assay was 93% (CI_95_ 87% – 96%) in RSV A and 96% (CI_95_ 89% – 99%) in RSV B positive samples. This difference was not statistically significant (*P* = 0.3, χ^**2**^-Test).

From the clinical perspective, *C*_*T*_ values were higher (and hence viral load lower) in respiratory specimens of older children and children admitted for non-respiratory reasons with concomitant respiratory tract infection (Additional file 1: Table S1). In consequence, the Alere i RSV sensitivity was 98% (CI_95_ 94% - 100%) in children <6 months, 94% (CI_95_ 81% - 99%) in children 6–11 months, 83% (CI_95_ 68% - 93%) in children 12–23 months and 87% (CI_95_ 73% - 96%) in children above 2 years of age (Table 3). In children hospitalized for URTI or LRTI, the Alere i RSV test sensitivity was 100% (CI_95_ 84–100%) and 96% (CI_95_ 92–99%), respectively. In children who were admitted for non-respiratory reasons, we found a sensitivity of 77% (CI_95_ 61% – 88%) (Table 3). As illustrated in Additional file 1: Figure S2, the relatively poor sensitivity in this group resulted from a high proportion of samples with low viral load (C_T_ value >30), and co-infection with *Influenza A, Parainfluenza, Rhinovirus* or *Coronavirus* was detected in 5 / 10 cases.

We did not undertake comprehensive analytical specificity testing but note that the 289 NPS with negative RT-PCR result included samples tested positive for *Influenza A, H1N1* or *B* (*n* = 42), *Rhinovirus* (*n* = 99), *Bocavirus* (*n* = 23), *Adenovirus* (*n* = 18), *Parainfluenza 1, 2, 3* or *4* (*n* = 36), *Coronavirus NL63*, *229E*, *OC43* or *HKU1* (*n* = 41), *Parechovirus* (n = 3), *Enterovirus* (n = 3), *Human metapneumovirus A/B* (*n* = 12) and *Mycoplasma pneumoniae* (n = 3). In RSV positive specimens, observed coinfections included *Rhinovirus* (*n* = 38), *Coronavirus* (*n* = 25), *Parainfluenza* (n = 12), *Bocavirus* (n = 9) and *Influenza* (*n* = 8). In 65 cases, neither RSV nor any of the above-mentioned pathogens were detected.

In our point-of-care setting, positive test results were identified after a mean duration of 5.1 min (CI_95_ 4.9–5.2 min; range 4.7–10.0 min). This included both sample pre-heating (3 min) and the amplification reaction. After the first testing, invalid results were reported in 6% (29/518). These NPS were directly retested, and valid (positive or negative) results were obtained in all cases.

## Discussion

We found that the novel Alere i RSV assay has a sensitivity of 93% (CI_95_ 89% – 96%) and a specificity of 96% (CI_95_ 93% – 98%) in a pediatric point-of-care setting. The test is user-friendly and test results are obtained in less than 13 min, with most positive test results being identified after approximately 5 min.

No direct comparison of Alere i RSV versus RADT was done in our study. Based on published data, the expected RADT sensitivity in young children is approximately 80% [9]. In our study setting, we previously found a RADT sensitivity of 63% (CI_95_ 55–72%) [14] and 55% (CI_95_ 45% – 64%) [15] over two different winter seasons. The Alere i RSV sensitivity is clearly superior to the expected RADT performance.

In children aged 24–35 months with lower viral load, the use of RADT is particularly limited with reported sensitivity of approximately 60% [16]. In patients ≥2 years of age, we found an Alere i sensitivity and specificity of 87% and 98% in comparison to our RT-PCR reference standard, respectively. The Alere i assay is suitable for point-of-care detection of RSV in children across all age groups.

Other sensitive rapid nucleic acid amplification assays are available for early detection of RSV infection. These assays require a testing time of at least 30 min to more than 1 h [17, 18]. Alere i RSV test results are available within 13 min, and positive test results are called out after a mean duration of approximately 5 min.

A limitation of our study is the use of VTM for Alere i RSV testing instead of directly inserting the swab to the Alere test base. Using VTM was required to establish the RT-PCR reference standard. This procedure is in accordance with the Alere i RSV package insert, but implies that samples are 1:5 diluted in comparison to directly inserting the swab. In our analysis, this could have provoked false negative results in samples with low viral load, and regular point-of-care users might prefer direct, non-diluted testing of swab specimens. Second, we compared Alere i RSV test results against a multiplex RT-PCR reference standard. Only divergent results were further evaluated by monoplex RT-PCR. Multiplex RT-PCR is usually slightly less sensitive than monoplex RT-PCR in samples with low viral load. [19] In pediatric patients, viral loads are usually high and in fact, 95% of our RSV positive samples had a *C*_*T*_ value <30. We therefore believe that multiplex RT-PCR is a rigorous reference standard in our study cohort, but acknowledge that applying a monoplex RT-PCR reference standard might have resulted in a slightly lower sensitivity of the Alere i RSV assay.

In summary, we evaluated the novel Alere i RSV assay in a pediatric emergency setting against a RT-PCR reference standard. The Alere i RSV performed well in the point-of-care setting, and sensitive test results were obtained across all pediatric age groups within 13 min. The assay requires a shorter test time than other currently available molecular test assays, and provides a significantly higher sensitivity than RADT assays.

## Conclusions

The Alere i RSV assay performs well in the pediatric point-of-care setting. The assay is easy to use, and the high sensitivity and specificity of test results help pediatricians to act appropriately both in patients with and without RSV infection.

## Additional file

Additional file 1: Supplementary Files (DOCX 206 kb)

## Abbreviations

altona RealStar PCR: altona RealStar RSV RT-PCR, altona Diagnostics, Hamburg, Germany
aRTI: acute respiratory tract infection
C_T_: cycle threshold
FTD 21: FTD respiratory pathogens 21 multiplex PCR
LRTI: lower respiratory tract infection
NPS: nasopharyngeal swabs
RADT: rapid antigen detection tests
RSV: Respiratory syncytial virus
RT-PCR: real-time reverse transcriptase polymerase chain reaction assay
URTI: upper respiratory tract infection
VTM: viral transport media
