# Supplementary material for: Performance of the Alere i RSV assay for point-of-care detection of respiratory syncytial virus in children
Source: BMC Infect Dis. 2017 Dec 13;17:767. doi: 10.1186/s12879-017-2855-1 (PMC5729395; doi:10.1186/s12879-017-2855-1)

**Supplementary Files**

Supplement 1 Mean FTD21 C_T_ values of RT-PCR positive samples by age group and admission diagnosis.

C_T_ values were significantly increased in older children (P<0.01, Kruskal-Wallis test) and in children with non-respiratory admission diagnosis (P<0.01, Kruskal-Wallis test).

|  | mean FTD21 C_T_ value (CI_95_) |
| --- | --- |
| **all RT-PCR positive samples** | 18.6 (17.8 – 19.3) |
| **admission diagnosis**  URTI  LRTI  non-respiratory | 20.0 (17.1 – 22.8)  17.7 (17.0 – 18.5)  21.1 (19.0 – 23.3) |
| **age group**  0 - 5 months  6 - 11 months  12 - 23 months  ≥ 2 years | 17.1 (16.2 – 17.9)  17.9 (16.4 – 19.5)  20.2 (18.1 – 22.2)  22.0 (19.7 – 24.2) |

**Supplement 2 C_T_ value, age, and Alere i test outcome in children with URTI, LRTI and non-respiratory admission diagnosis.**

The scatter plot illustrates the FTD21 C_T_ value_,_ age, and Alere i test outcome in children hospitalized
A) for URTI; B) for LRTI and C) for non-respiratory reasons with concomitant aRTI.

Black: Sample with true positive Alere i test result; Red: Sample with false negative Alere i test result


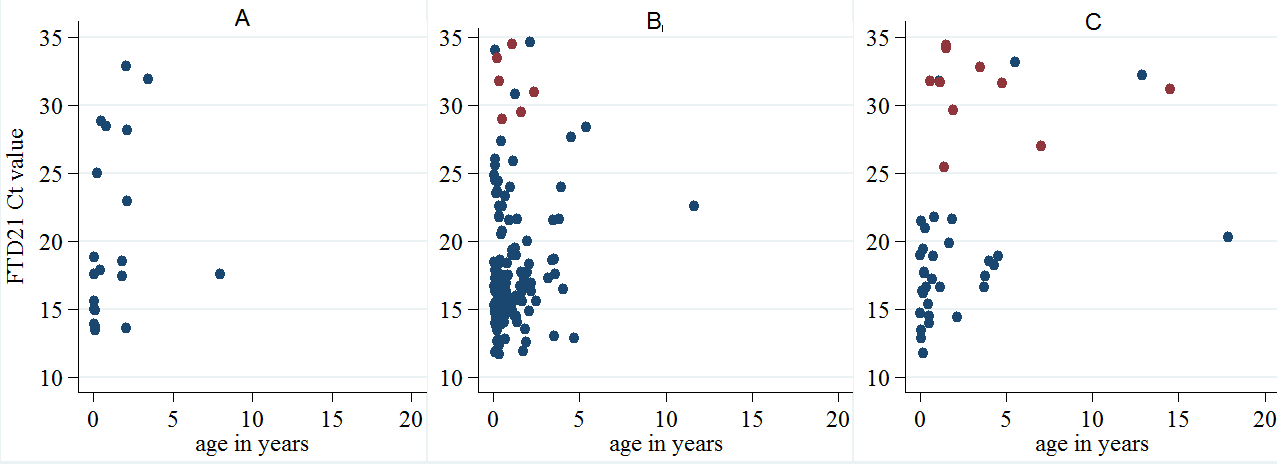


**Supplement 3 RT-PCR reference testing**

Initial FTD21 analysis was conducted on 518 NPS included in the analysis. In 37 cases (12 FTD21 negative and 25 FTD21 positive samples), the multiplex FTD21 outcome did not correspond to the Alere i RSV test results. Independent aliquots of these samples were analyzed by monoplex RT-PCR (altona RealStar). The RealStar results confirmed the previous FTD21 result in 25/ 37 cases, including 11 and 15 samples which were considered false positive or false negative. In 12 samples, test results differed between the two RT-PCR assays. These samples were again tested by the FTD21 assay. This second FTD21 analysis corresponded to the altona RealStar result in 10 NPS (1 true positive samples, 9 true negative samples).

In one sample, repeated FTD analysis was not possible due to insufficient sample volume. We conservatively considered this sample to be “false negative”.


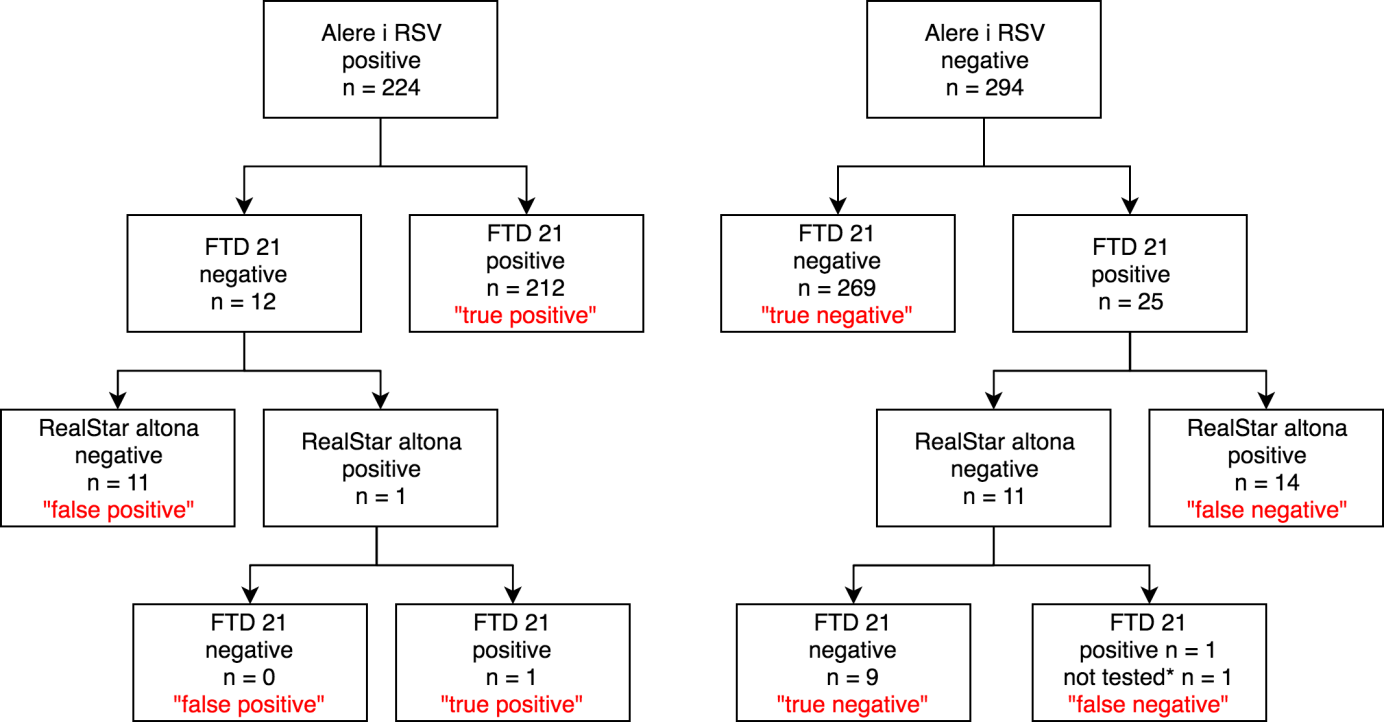

Supplement: Additional file 1: — Supplementary Files (DOCX 206 kb) [file 12879_2017_2855_MOESM1_ESM.docx]
